# A Literature Review of the Renal Manifestations in Pediatric Celiac Disease and Its Associated Clinical Implications

**DOI:** 10.7759/cureus.99773

**Published:** 2025-12-21

**Authors:** Ragad El Ballushi, Meriem Akeblersane, Nairouz Quateen, Ahmed Elballushi, Tala Alul, Arun Nair

**Affiliations:** 1 School of Medicine, Royal College of Surgeons in Ireland - Bahrain, Muharraq, BHR; 2 Pediatric Hematology Oncology, Cohen Children's Medical Center, New Hyde Park, USA

**Keywords:** celiac disease, malnutrition, pediatric gastroenterology, pediatrics, renal complications

## Abstract

Celiac disease (CeD) is a chronic autoimmune disorder predominantly targeting the small intestine, in which the immune system reacts to gluten. It is increasingly recognized as a systemic condition with potential renal involvement and is observed in children worldwide. There have been reports of renal manifestations in children with CeD across Europe, North Africa, the Middle East, and parts of Asia, including immunoglobulin A (IgA) nephropathy (IgAN), diabetic nephropathy, and urolithiasis. These conditions are frequently associated with immune complex deposition, persistent inflammation, and nutritional deficits caused by malabsorption. Despite increased recognition, research in pediatric populations remains limited, and screening and management practices are inconsistent. This study identifies renal impairment as an underrecognized extraintestinal manifestation of CeD in the pediatric population, emphasizing the protective role of an early gluten-free diet and summarizing current evidence on mechanisms and clinical presentation to guide future pediatric-focused research.

## Introduction and background

Celiac disease (CeD) is an autoimmune-mediated enteropathy affecting genetically susceptible individuals, in whom ingestion of gluten, a protein found in wheat, barley, and rye, triggers immune-mediated injury to the small intestine [1]. Traditionally, clinicians recognized CeD by its gastrointestinal manifestations such as diarrhea, abdominal distension, and unexplained weight loss. However, it is now well established as a systemic disorder with extraintestinal and multisystem involvement, including renal complications [2].

The small bowel villi play a central role in nutrient absorption, and their destruction explains malabsorptive symptoms. Beyond this, CeD is increasingly understood as a systemic immune disease rather than a purely intestinal condition. Among the extraintestinal complications, renal involvement has been reported more frequently, although guidelines remain limited and routine screening recommendations are lacking. The most documented renal manifestations in pediatric celiac disease include immunoglobulin A (IgA) nephropathy (IgAN), which is globally the most common form of glomerulonephritis, as well as membranous nephropathy, nephrotic syndrome, and interstitial nephritis [3].

The underlying mechanisms, clinical consequences, and potential importance of early dietary intervention are highlighted in this overview of the renal manifestations of pediatric CeD, with a focus on IgAN, diabetic kidney disease, and urolithiasis. The possible risks of renal manifestations are particularly significant in children, whose immune and physiological systems are still developing. Understanding these renal associations offers opportunities for earlier diagnosis, timely treatment, and improved long-term outcomes [4]. This study will further explore pediatric involvement in these renal manifestations, discuss the existing gaps in the literature, and highlight the importance of future studies to better understand and address these associations in the pediatric population.

## Review

Pathophysiology 

According to recent research, people with CeD are more likely to have renal diseases, such as chronic kidney disease (CKD) [5]. CeD and renal symptoms have a complicated association, including immune-mediated processes, long-term inflammation, and malabsorption-related deficiencies [5-7]. Growing evidence supports the existence of a gut-kidney axis in which increased intestinal permeability allows bacterial byproducts such as lipopolysaccharides to enter the circulation, leading to systemic inflammation and renal injury [8]. These mechanisms may amplify the renal susceptibility already imposed by circulating immune complexes and systemic autoimmune activation [6].

Persistent immune activation is the central driver of disease progression in celiac disease. Consuming gluten produces anti-tissue transglutaminase IgA autoantibodies and has been shown to lead to systemic consequences. IgA immune complexes can deposit within the glomerular mesangium and trigger a cascade of hematuria, proteinuria, and progressive chronic kidney disease [8,9]. Although less common, other nephropathies, such as interstitial nephritis and membranous nephropathy, are nonetheless significant. The precise molecular links are not fully understood, though hypotheses include systemic inflammation, molecular mimicry between gut and kidney antigens, and cross-reactivity of dietary determinants with renal targets. Interestingly, anti-tissue transglutaminase antibodies, primarily associated with small bowel pathology, have also been identified in renal vascular regions, suggesting a potential direct histological role in kidney injury [8].

Another vital mechanism involves nutritional deficiencies due to malabsorption. Vitamin D insufficiency can cause calcium-phosphate imbalance, leading to nephrocalcinosis or secondary hyperparathyroidism [10]. Chronic hypoalbuminemia brought on by protein-losing enteropathy can alter glomerular filtration and oncotic pressure [11]. According to clinical research, early adoption of a gluten-free diet may help prevent or reverse kidney damage and lower antibody titers in affected children [12].

Immune complex depositions

CeD is an immune-mediated enteropathy triggered by gluten ingestion, producing autoantibodies against tissue transglutaminase [13]. CeD and IgAN share a strong correlation, as both conditions stem from a common autoimmune origin. IgAN is the most frequently reported renal disorder in CeD, with studies suggesting a threefold increased risk compared to the general population [14,15]. It is believed that aberrant IgA synthesis in response to gluten causes immune complex accumulation in the kidneys and glomerular damage, which in turn causes IgAN and CeD [16]. This process involves pro-inflammatory cytokine release and immune cell activation, causing kidney inflammation and damage [17]. Additionally, the interaction of polymeric IgA1 with the TfR1/CD71 receptor on mesangial cells further promotes inflammation and may contribute to broader kidney injury [18]. CeD-associated immune dysregulation leads to circulating IgA1-containing immune complexes that deposit in the renal mesangium, triggering complement activation, cytokine release, mesangial proliferation, and fibrosis [19-21]. Clinically, IgAN manifests with hematuria, proteinuria, and progressive renal insufficiency. Activation of the renin-angiotensin and complement systems accelerates the development of tubulointerstitial fibrosis and glomerulosclerosis, ultimately decreasing renal function [19]. This clinical link emphasizes the importance of recognizing renal manifestations in celiac disease and highlights the need for further research into shared pathogenic mechanisms and potential therapeutic strategies. Given the limited pediatric-specific data, applying these insights to understand renal involvement in children with CeD is a critical research priority, particularly to elucidate early immune alterations and their long-term renal consequences in this population [8].

Membranous glomerulonephritis

Though less frequent, membranous glomerulonephritis (MGN) has also been reported in CeD. Although the exact mechanism is unclear, chronic immune activation and autoantibody production are suspected contributors [22]. MGN is characterized by subepithelial immune complex deposits and the glomerular basement membrane (GBM) thickening, often resulting in nephrotic syndrome [21,22]. According to some evidence, a gluten-free diet (GFD) may help MGN even if the literature is confined to case studies [23]. Renal tissue has even been found to have TG2 antibodies. These results emphasize how crucial it is to rule out other etiologies and consider CeD a possible secondary cause of MGN. Although uncommon, minimal change disease (MCD) and focal segmental glomerulosclerosis (FSGS) have also been reported in CeD [24-26]. Their co-occurrence underscores potential shared autoimmune mechanisms, though evidence remains scarce.

Chronic inflammation

Another possible mechanism of renal injury in celiac disease is chronic systemic inflammation, which can disrupt the endothelium's protective functions and trigger endothelial dysfunction in the kidney [27]. This dysfunction involves dysregulation of the complement system, driving excessive inflammation and immune-mediated glomerular damage, overactivity of the renin-angiotensin system (RAS), which encourages pro-inflammatory signaling, oxidative stress, and vasoconstriction, and disturbances in coagulation and angiogenesis, resulting in increased permeability, microvascular thrombosis, and poor vascular healing. Combined, these mechanisms weaken the glomerular filtration barrier, leading to fibrosis, inflammation, proteinuria, and a gradual decline in kidney function [27].

Nutritional implications 

Malabsorption contributes significantly to renal complications in CeD. Vitamin D, calcium, magnesium, and iron deficiency disrupt mineral and hematologic balance, predisposing to nephrolithiasis, nephrocalcinosis, anemia, and secondary hyperparathyroidism. Vitamin D deficiency disrupts calcium-phosphorus metabolism, contributing to nephrolithiasis and renal tubular injury [28]. Iron deficiency anemia, the most common extraintestinal manifestation of CeD, worsens CKD-related anemia [29,30]. Calcium and magnesium deficiency promote secondary hyperparathyroidism and impaired PTH function, with downstream renal consequences [31]. Enteric hyperoxaluria in untreated CeD increases nephrolithiasis risk, especially in adults. In children, urinary calcium excretion is often low, though oxalate-driven stone risk persists [32,33]. Unabsorbed fatty acids bind luminal calcium, leaving oxalate free for colonic absorption; bile acids further increase colonic permeability, amplifying hyperoxaluria [34,35]. The absorbed oxalate is excreted in urine, promoting calcium oxalate crystallization and, in severe cases, oxalate nephropathy [34]. Early recognition of malabsorption and institution of a GFD with adequate calcium and a reduced oxalate diet (and citrate optimization as needed) are essential to mitigate kidney injury.

Discussion

CeD is a chronic immune-mediated enteropathy with multisystemic manifestations beyond the gastrointestinal tract, including renal involvement, which is increasingly recognized as clinically significant in children. Growing evidence supports the existence of a gut-kidney axis in which increased intestinal permeability allows bacterial byproducts such as lipopolysaccharides to enter the circulation, leading to systemic inflammation and renal injury [8]. This further highlights the need for long-term, multidisciplinary follow-up that includes renal monitoring as part of routine CeD care.

Although the absolute incidence of renal disease in pediatric CeD is low, several studies report a higher relative risk of chronic kidney disease (CKD), glomerulonephritis, and end-stage renal disease compared with the general population [8], of which the common presentations include IgAN and diabetic nephropathy. Current screening recommendations remain divided, where some guidelines support universal renal monitoring, and others restrict it to high-risk subgroups such as those with type 1 diabetes or persistent celiac seropositivity [12,36]. The absence of consensus underlines the crucial need for standardized pediatric protocols.

IgAN is most frequently associated with CeD, as mentioned prior. Both diseases share immunogenetic pathways involving mucosal immune activation and increased intestinal permeability [12,15]. Although only a weak association was described in the literature, it is imperative to note that case reports describe stabilization of renal function and resolution of hematuria following GFD initiation [12]. Such cases suggest that gluten-driven immune activation can possibly lead to glomerular injury in susceptible children. Diabetic nephropathy represents another critical overlap.

The reported prevalence estimates suggest that type 1 diabetes mellitus (T1DM) coexists with CeD in approximately 3%-12% of children, although this range varies widely across studies [4,8]. Studies reveal a higher incidence of microalbuminuria in patients with both disorders, independent of glycemic control [37]. However, additional factors that may worsen renal injury include prolonged disease duration, nutritional deficiencies, and hyperhomocysteinemia [38]. Early CeD screening and GFD adherence in children with T1DM may therefore mitigate cumulative kidney risk. Evidence for urolithiasis in pediatric CeD is conflicting. While adults show increased urinary oxalate and stone risk, pediatric studies have demonstrated normal oxalate but low urinary calcium levels, implying a potentially reduced risk [8,39]. Given this variability, screening for nephrolithiasis should be individualized. Importantly, reports of renal manifestations in pediatric CeD originate from geographically diverse regions, including Europe, North Africa, the Middle East, and parts of Asia, highlighting that these associations are recognized across multiple populations. Differences in screening practices, serologic testing availability, environmental exposures, and genetic susceptibility likely contribute to regional variation in the reported burden of renal disease [4,8]. Overall, differences in diagnostic criteria, study design, and follow-up duration highlight the need for large, prospective pediatric studies.

Historical trends have demonstrated how environmental and dietary exposures can affect disease expression. Interestingly a surge in CeD diagnoses in the 1980s in Sweden was noted to be due to high gluten content in infant foods which raised the question whether gluten timing or dose affects disease risk [40]. Subsequent trials found no consistent association between age of introduction and CeD development [41,42], though dose-dependent mucosal injury from gluten may explain extraintestinal complications, including renal inflammation [38,43].

Routine renal assessment for pediatric CeD has been recommended to include urinalysis, serum creatinine, and eGFR [44]. The urinary albumin-to-creatinine ratio offers a sensitive early marker of kidney injury [44], while ultrasonography and advanced imaging help identify structural or complex abnormalities [45]. These non-invasive tools can facilitate earlier detection and timely intervention.

Specific hospitalization data for renal complications are lacking, though studies show that CeD diagnosis and GFD initiation reduce overall healthcare utilization and infection-related admissions [46-48]. No pediatric cases of renal transplantation directly attributable to CeD have been reported, which does show the rarity of fulminant renal complications; however, one must consider under-reporting/under-recognition of the condition as well [49]. These gaps highlight the need to quantify the burden of renal involvement and long-term outcomes.

The GFD remains the cornerstone of CeD therapy and the only intervention shown to reverse systemic complications. Early adherence in children can aid in improving bone density and reduce albuminuria, suggesting reversible renal injury before permanent glomerular damage occurs [36,50]. Nonetheless, adherence to the diet is a complication that requires focus, especially among adolescents who face social pressures, stigma, and nutritional imbalances, and can be addressed in a multidisciplinary approach [42,51].

Emerging treatments such as larazotide acetate, which enhances intestinal tight-junction integrity, and the peptide-based vaccine Nexvax2 offer promising future direction [29]. While current trials are limited to adults, extending these investigations to children could potentially provide insights into the effects on immune modulation and renal outcomes. To understand the current burden of renal disease in this population, Table 1 summarizes current quantitative evidence on the prevalence, incidence, and relative risk of renal outcomes in patients with celiac disease, highlighting clinical significance for early detection and management. The data indicates a persistently increased risk of renal manifestations, emphasizing the importance of early renal screening and intervention in clinical practice.

**Table 1:** Reported Incidence and Risk of Renal Manifestations in Celiac Disease IgA: Immunoglobulin A; ESRD: end-stage renal disease; GN: glomerulonephritis; CGN: cresentic glomerulonephritis; HR: hazard ratio; OR: RR: relative risk; OR: odds ratio

Study (Year)	Renal Outcome	Measure	Incidence / Prevalence	Key Result (95% CI, p-value)	Interpretation / Clinical Significance
The Risk of Renal Comorbidities in Celiac Disease Patients Depends on the Phenotype of CD [44]	IgA nephropathy	HR	0.56% in CD vs 0.03% in controls	HR=18.98 (2.29-157.63), p=NR	Strong association suggesting immune-mediated glomerular injury.
Coeliac Disease and Risk of Renal Disease: A General Population Cohort Study [52]	GN	HR	89 events	HR=1.64 (1.01-2.66), p=0.046	Moderate risk increase.
Coeliac Disease and Risk of Renal Disease: A General Population Cohort Study [52]	CGN	HR	39 events	HR=2.65 (1.34-5.24), p=0.005	Moderate risk increase.
Coeliac Disease and Risk of Renal Disease: A General Population Cohort Study [52]	Dialysis	HR	102 events	HR=3.48 (2.26-5.37), p<0.001	Higher risk of severe renal outcome.
Increased risk of end-stage renal disease in individuals with coeliac disease [53]	ESRD	HR	90 vs 31 expected	HR=2.87 (2.05-4.03), p<0.001	Threefold higher ESRD risk.
Celiac Disease and Risk of Kidney Diseases: Meta-analysis [5]	Any kidney disease	RR	—	RR = 2.01 (1.31-3.09), p<0.01	Confirmed elevated renal risk.
Celiac Disease and Risk of Kidney Diseases: Meta-analysis [5]	IgAN	RR	—	RR=2.62 (1.84-3.72)	Significant association.
Celiac Disease and Risk of Kidney Diseases: Meta-analysis [5]	ESRD	RR	—	RR=2.57 (1.38-4.78)	Increased ESRD risk.
Association Between Celiac Disease and Risk of Kidney Disorders [54]	Kidney disease	OR	2.29%	OR=1.98 (1.62-2.41), p<0.001	Consistent pattern of renal risk.
Association Between Celiac Disease and Risk of Kidney Disorders [54]	GN	OR	1.31%	OR=2.68 (1.80-3.99)	Significant association.
Association Between Celiac Disease and Risk of Kidney Disorders [54]	ESRD	OR	1.55%	OR=2.58 (1.65-4.02)	Increased ESRD risk.

Table 2 summarizes current clinical recommendations for renal screening and management in pediatric and mixed celiac disease cohorts. It highlights how early identification and intervention for renal complications, based on both guidelines and case reports, may improve outcomes while emphasizing gaps in pediatric-specific evidence. 

**Table 2:** Renal Screening Recommendations, Interventions, and Evidence Gaps in Celiac Disease Cohorts with Emphasis on Pediatric Findings CeD: Celiac disease; ESPGHAN: European Society for Pediatric Gastroenterology, Hepatology and Nutrition; GN: glomerulonephritis; ACEi: angiotensin-converting enzyme inhibitor; ACR: albumin creatinine ratio; IgAN: IgA nephropathy; RTA: renal tubular acidosis

Paper (Cohort)	Recommendation for Renal Screening	Intervention / Management	Notes / Gaps	Citation
Mixed cohort	Screen for haematuria/proteinuria and check renal function if abnormal labs.	Initiate gluten-free diet (GFD).	Observational evidence only; limited pediatric data.	[54]
Mixed cohort	If abnormal urinalysis/proteinuria → quantify ACR/24-h and refer to nephrology.	Manage GN with ACEi or immunosuppression as appropriate.	Need pediatric studies to define screening thresholds.	[54]
ESPGHAN (pediatric)	No routine renal screening; assess if extraintestinal signs (growth failure, anaemia, oedema).	Perform renal workup if indicated; standard GFD therapy.	Gap: lack of formal pediatric renal surveillance guidance.	[55]
Pediatric case (IgAN+CeD)	Screen CeD patients for haematuria/proteinuria.	Coordinate with nephrology; treat with GFD and ACEi.	Evidence limited to case reports; causality unclear.	[12]
Pediatric case (distal RTA)	Test CeD patients with unexplained RTA for serology.	Give bicarbonate/potassium and start GFD.	Only case-level data; need series confirmation.	[56]
Pediatric case (urolithiasis)	Test for CeD in recurrent nephrolithiasis with GI symptoms.	Start GFD and manage stones per standard care.	Mechanistic link hypothesized; evidence anecdotal.	[57]

## Conclusions

In summary, renal involvement in pediatric CeD is a clinically relevant extraintestinal symptom that is often overlooked. With differing degrees of evidence, IgA nephropathy, diabetic kidney disease, and urolithiasis are the most often documented renal consequences. Early adoption of a gluten-free diet lowers systemic immunological activation and may eventually assist in maintaining kidney function; however, the precise processes are still unclear. These findings support the necessity for a more comprehensive strategy to manage pediatric CeD. Beyond gastrointestinal symptoms, routine follow-up should involve renal evaluation using blood pressure monitoring, kidney function tests, and urinalysis. Closer monitoring is necessary for children with comorbid conditions like type 1 diabetes since they are more likely to develop nephropathy. Immune dysregulation, intestinal barrier dysfunction, and dietary deficits are all part of CeD, and they may eventually lead to kidney damage. Because it may lessen intestinal and systemic effects, strict adherence to a gluten-free diet is still necessary. There are still significant research gaps, nevertheless. It is now challenging to make definitive conclusions because the majority of the evidence comes from adult research or tiny pediatric cohorts. To elucidate prevalence, causes, and outcomes, larger longitudinal pediatric research is required. Children with CeD can have better long-term kidney health and a better overall prognosis with early detection and aggressive renal monitoring.
